# Intervention Efficacy of Slightly Processed Allergen/Meat in Oral Immunotherapy for Seafood Allergy: A Systematic Review, Meta-Analysis, and Meta-Regression Analysis in Mouse Models and Clinical Patients

**DOI:** 10.3390/nu16050667

**Published:** 2024-02-27

**Authors:** Xinyu Han, Xinya Wang, Xiaotong Chen, Hong Liu, Jingwen Liu, Mary Miu Yee Waye, Guangming Liu, Shitao Rao

**Affiliations:** 1 College of Ocean Food and Biological Engineering, Xiamen Key Laboratory of Marine Functional Food, Fujian Provincial Engineering Technology Research Center of Marine Functional Food, Jimei University, Xiamen 361021, China; 201914908009@jmu.edu.cn (X.H.); liuhong@jmu.edu.cn (H.L.); jwliu@jmu.edu.cn (J.L.); 2 Department of Bioinformatics, Fujian Key Laboratory of Medical Bioinformatics, Institute of Precision Medicine, School of Medical Technology and Engineering, Fujian Medical University, Fuzhou 350122, China; 1306234259@fjmu.edu.cn (X.W.); chenxt@fjmu.edu.cn (X.C.); 3 The Nethersole School of Nursing, Croucher Laboratory for Human Genomics, The Chinese University of Hong Kong, Shatin, N.T., Hong Kong SAR, China; mary-waye@cuhk.edu.hk; 4 School of Biomedical Sciences, The Chinese University of Hong Kong, Shatin, N.T., Hong Kong SAR, China

**Keywords:** fish and crustacean allergens, OIT, anaphylactic score, incidence rate ratio, processing methods

## Abstract

**Background**: Seafood allergy is a significant global health concern that greatly impacts a patient’s quality of life. The intervention efficacy of oral immunotherapy (OIT), an emerging intervention strategy, for seafood allergy remains controversial. This study aimed to perform a systematic review and meta-analysis to evaluate the efficacy of slightly processed allergen/meat from fish and crustacea in OIT, both in mouse models and clinical patients. **Methods**: A comprehensive literature search was performed in four mainstream databases and the EBSCOhost database to identify all relevant case–control and cohort studies. The aim was to elucidate the intervention efficacy, encompassing various processing methods and assessing the efficacy of multiple major allergens in OIT. **Results**: The meta-analysis included five case–control studies on crustacean allergens in mouse models and 11 cohort studies on meat from fish and crustacea in clinical patients for final quantitative assessments. In mouse models, crustacean allergen substantially decreased the anaphylactic score after OIT treatment (mean difference (MD) = −1.30, *p* < 0.01). Subgroup analyses with low-level heterogeneities provided more reliable results for crab species (MD = −0.63, *p* < 0.01, I^2^ = 0), arginine kinase allergen (MD = −0.83, *p* < 0.01, I^2^ = 0), and Maillard reaction processing method (MD = −0.65, *p* < 0.01, I^2^ = 29%), respectively. In clinical patients, the main meta-analysis showed that the slightly processed meat significantly increased the incidence rate of oral tolerance (OT, incidence rate ratio (IRR) = 2.90, *p* < 0.01). Subgroup analyses for fish meat (IRR = 2.79, *p* < 0.01) and a simple cooking treatment (IRR = 2.36, *p* = 0.01) also demonstrated a substantial increase in the incidence rate of OT. Sensitivity and meta-regression analyses successfully identified specific studies contributing to heterogeneity in mouse models and clinical patients, although these studies did not impact the overall significant pooled effects. **Conclusions**: This meta-analysis provides preliminary evidence for the high intervention efficacy of slightly processed allergen/meat from fish and crustacea in OIT, both in mouse models and clinical patients. The Maillard reaction and cooking processing methods may emerge as potentially effective approaches to treating allergen/meat in OIT for clinical patients, offering a promising and specific treatment strategy for seafood allergy. However, these findings should be interpreted cautiously, and further supporting evidence is necessary.

## 1. Introduction

Food allergy is a global health concern and affects approximately 2–5% of adults and 5–8% of children worldwide [1]. Individuals with allergies may experience gastrointestinal discomfort, urticaria, wheezing, shortness of breath, or even life-threatening anaphylactic reactions [2]. The underlying mechanism of the disease involves the production of specific immunoglobulin E (IgE) in response to particular allergens present in various types of foods. The World Health Organization/Food and Agriculture Organization has identified eight major allergenic food categories, including eggs, milk, peanuts, sesame seed, nuts, wheat, fish, and crustaceans [3]. More recently, mollusks have been added to the ‘Priority Allergic Food List’ in the European Union and Asian regions [4]. Unlike allergies to eggs or milk, which tend to improve with age, allergic reactions caused by aquatic products such as fish, crustaceans, and mollusks often persist throughout a person’s lifetime, leading to chronic anxiety and significantly impacting their quality of life [5].

The conventional management approach to reducing the occurrence rate of seafood allergy involves strict avoidance of allergic foods, both through consumption and physical contact. However, a long-term avoidance of allergic foods would cause adverse consequences, such as severe adverse reactions upon accidental ingestion and the potential development of protein–energy malnutrition or even mental illnesses [6]. Furthermore, with the rapid development of the seafood industry, numerous aquatic foods are now widely used as ingredients and additives in daily life, leading to an increase in accidental ingestion cases [7]. Notably, there have been reported cases of individuals experiencing atopic dermatitis after coming in contact with fish [8]. These findings highlight the growing diversification and severity of food allergies, necessitating the development of specific and effective strategies beyond simple avoidance methods. While biological monotherapy, such as omalizumab, has demonstrated efficacy in treating food allergy, its high cost limits its wide adoption [9,10]. Therefore, there is an urgent need for a more affordable and practical approach to overcome these challenges.

Oral immunotherapy (OIT) is emerging as a promising alternative strategy for managing food allergies by suppressing the pathogenic immune response mediated by allergen-specific IgE. Desensitization treatment involves gradually exposing allergic patients to increasing small doses of the offending foods, allowing for the prevention of severe clinical symptoms and the enhancement of immune regulatory mechanisms [11,12,13]. The approach has shown success in treating peanut allergies, where allergen-specific CD4+ T cells transition into an unresponsive Th2 phenotype [14]. The approval of the peanut protein extract Palforzia for OIT by the Food and Drug Administration in 2020 was a significant milestone. However, the financial burden associated with Palforzia poses a challenge for patients. Additionally, mild to severe adverse reactions are commonly observed during treatment periods, affecting multiple tissues such as gastrointestinal and respiratory tracts [15]. Wongteerayanee et al. observed a high rate of adverse effects in the OIT protocol for highly wheat-sensitized patients [16]. Palosuo et al. observed that approximately 82% of children experienced gastrointestinal symptoms during egg OIT [17]. The use of unprocessed allergic foods or extracted allergens in OIT is hindered by their inherent allergenicity, which can lead to anaphylaxis and contribute to the high rate of clinical adverse reactions, including angioedema, diarrhea, and urticaria [18]. Consequently, there is a pressing need for safer OIT products and the exploration of alternative patient groups and food allergens [19].

Several slight processing methods have been proposed to reduce the allergenicity of raw foods while maintaining their oral tolerance (OT) potential. These methods include heating, boiling, Maillard reaction (MR), enzymatic cross-linking, and enzymatic degradation [20,21,22,23]. For example, Saifi et al. demonstrated the safety of cooked eggs in OIT, reporting a very low reaction rate (1.8%) of mild adverse events [24]. Baked milk has also been shown to be safer than raw milk in most cases of OIT, particularly for individuals with severe allergic reactions to unprocessed milk [24,25]. Furthermore, the inclusion of egg and peanuts in muffins has shown potential in promoting tolerance to food allergens [26]. Regarding slightly processed seafood allergens, there have been a limited number of studies on desensitization treatment using OIT. However, the findings from these studies remain controversial and subject to ongoing debate [27,28,29]. Liu et al. conducted research on enzymatic cross-linked crab tropomyosin (TM) with horseradish peroxidase and observed a substantial reduction in the allergenicity of the allergen while maintaining its OT potential [27]. It is worth noting that the glycation of shrimp TM with glucose, maltotriose, maltopentaose, and maltoheptaose showed a significant decrease in IgE reactivity, except for TM-maltose, which exhibited increased IgE reactivity possibly due to the formation of advanced glycation end products (AGEs) as neoallergens [30]. Furthermore, glycation may have varying effects on allergenicity in TM for different species within the shellfish family [31]. Overall, the complex relationship between processed seafood products, allergenicity, and OT potential is influenced by various confounding factors, making it difficult to draw conclusive results.

In this study, we conducted a comprehensive literature search to identify eligible studies for a systematic review and a further quantified meta-analysis, aiming to clarify the ambiguous correlations between slightly processed allergen/meat from fish and crustacea and their OT potential. The relationship between processing methods and food allergenicity is a topic of ongoing controversy, and there is a significant gap between allergenicity and OT potential [19]. Our meta-analyses aimed to include all published studies that compared the intervention efficacies of different processing methods. We included both clinical patients undergoing desensitization treatment and mouse models, which are more readily available for research purposes. We focused on the major allergens for crustacea (TM; arginine kinase, AK) and fish (parvalbumin, PV) as the specific allergens of interest. In addition to the main meta-analyses, we conducted several subgroup analyses based on various types of processing methods, seafood species, and major allergens. This approach allowed us to delve deeper into the specific factors that may influence the OT potential of slightly processed seafood allergens/meat.

## 2. Materials and Methods

### 2.1. Search Strategy

**Figure 1:**
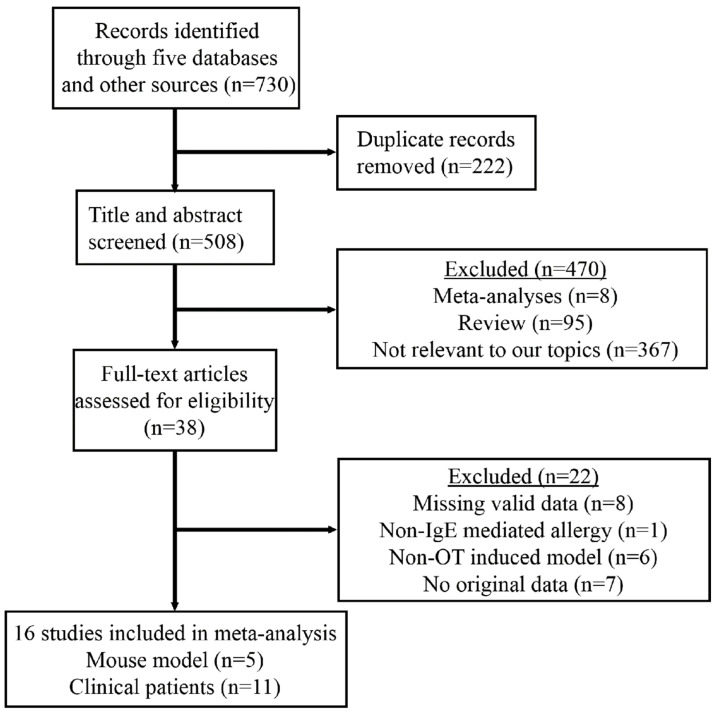
The PRISMA flow diagram for selection procedures of studies. PRISMA: Preferred Reporting Items for Systematic Reviews and Meta-Analyses.

As of December 2022, a systematic search was conducted to identify case–control and cohort studies on the effects of slightly processed food in OIT. The search was performed in four mainstream databases: PubMed, Ovid, Web of Science, and Cochrane Library, and a comprehensive EBSCOhost database, which provides access to Academic Search Premier, CINAHL Complete, eBook Collection, EBSCO eClassics Collection, MEDLINE, APA PsycInfo, and Library, Information Science & Technology Abstracts. In order to include all potentially relevant studies, the reference lists of relevant reviews and conference proceedings were also searched (Figure 1). Notably, the literature search was limited to articles in the English language.

The electronic search formula was defined as follows: (‘food allergen’ OR ‘food allergy’ OR ‘allergenicity’ OR ‘allergen’ OR ‘food hypersensitivity’) AND (‘oral tolerance’ OR ‘oral immunotherapy’ OR ‘tolerance’) AND (‘processing’ OR ‘Maillard’ OR ‘enzymatic’ OR ‘heating’ OR ‘canning’ OR ‘pressure’ OR ‘baking’ OR ‘base’ OR ‘glycosylation’ OR ‘deglycosylation’ OR ‘acid’ OR ‘ultrasonic’ OR ‘irradiation’ OR ‘fermentation’ OR ‘thermal’ OR ‘boil’ OR ‘steam’ OR ‘microwave’ OR ‘treatment’) AND (‘fish’ OR ‘shellfish’ OR ‘mollusks’ OR ‘shrimp’ OR ‘crab’ OR ‘scallop’ OR ‘oysters’ OR ‘abalone’ OR ‘aquatic product’ OR ‘seafood’ OR ‘tropomyosin’ OR ‘arginine kinase’ OR ‘sarcoplasmic calcium binding protein’ OR ‘PV’ OR ‘parvalbumin’ OR ‘TM’ OR ‘AK’ OR ‘SCP’ OR ‘cod’ OR ‘crayfish’ OR ‘lobster’ OR ‘crawfish’). Two authors independently screened the articles to determine whether they met the inclusion criteria outlined below. The initial screening involved reviewing the titles and abstracts of the articles. Subsequently, the full texts of the selected articles were assessed to determine their eligibility for inclusion in the final analysis. In cases where inconsistencies arose, the authors engaged in discussion to reach a consensus. Ultimately, eligible articles were identified for inclusion in the quantitative analysis.

### 2.2. Eligibility Criteria

*Inclusion criteria:*

(1) Case-control, case study, and cohort studies.
(2) OIT experiments conducted in either mouse models or clinical patients.
(3) Mouse models: The primary indicator is the change in anaphylactic score before and after OIT treatment as the score integrated the allergic symptoms of mouse (e.g., body temperature reduction, diarrhea rate) and biological markers (e.g., IgE, IgG1, IgG2a, histamine).
(4) Clinical patients: The primary indicator of OIT intervention efficacy is the successful tolerance rate determined through the double-blind oral challenge. This rate directly signifies the achievement of a desensitized state by patients during the maintenance phase under these conditions. Additionally, it is worth noting that the drop-out rate is another crucial indicator when evaluating the efficacy of OIT intervention.
(5) Availability of data before and after OIT treatment.
(6) Additional evaluation indicators in both mouse models and clinical patients including specific antibodies (IgE), expression levels of various cytokines (IL-4, IL-10, IL-13, TGF-β and INF-γ), main effector cell clusters in mouse models, and OT dosage of meat and wheal size in clinical patients.

*Exclusion Criteria:*

(1) Non-original article (e.g., narrative reviews, editorials, meta-analyses).
(2) Experiments conducted solely on cell models.
(3) Allergic reactions not mediated by the IgE.
(4) Mouse sensitization model not constructed by the oral gavage route. Evaluation indicators for slightly processed food in patients limited to allergenicity, without assessing their potential for OT.
(5) Missing data either before or after OIT treatment.
(6) Unobtainable raw data.

### 2.3. Data Extraction

Data extraction was conducted by one researcher, and another researcher reviewed and verified the extracted data. The extracted information from both mouse-model and clinical studies included the following: first author, year of publication, study design (parallel and crossover experiments), number of mice/patients included, type of allergen, methods used for processing, main indicators, and other relevant indicators. In studies involving mouse models, if available, the anaphylactic scores before and after OT were extracted from each mouse. If the anaphylactic score was not reported, the mean and standard deviation statistics were directly obtained. For clinical studies involving allergic patients, the incidence rates of OT challenge before and after treatment were extracted for each participant. In studies that evaluated tolerance dosages at multiple time points, the dosage during the maintenance phase, rather than the build-up phase, was utilized for further analyses. In cases where studies recruited fewer than three patients, those studies were combined into one effective study if a low heterogeneity was observed among them. This approach was adopted to avoid potential bias resulting from very small sample sizes in individual studies.

### 2.4. Quality Assessment

The quality assessment of all included studies was conducted using the Newcastle–Ottawa Scale (NOS), a widely utilized tool for assessing case–control and cohort studies. For the case–control studies conducted in mouse models, the evaluation focused on the selection of cases and controls, comparability of cases and controls based on design or analysis, and ascertainment of exposure. Due to the differences between mouse models and clinical patients, one item in the original scale, ‘representativeness of the cases’, was modified to ‘application of correct mouse model’ in order to better reflect the quality of the included studies in mouse models. In the case of cohort studies involving clinical patients, the emphasis was on the selection of exposed and non-exposed cohorts, comparability of cohorts, and assessment of outcome. After calculation, stars of 7~9 represented a high quality, 5~6 stars represented a moderate quality, and 0~4 stars represented a low quality. Two independent authors initially evaluated the quality for all included studies, and any disagreement was resolved through discussion or consultation with a senior author if necessary.

### 2.5. Meta-Analysis

The statistical analyses were conducted using the ‘meta’ package in the R programming language (version 4.0.0) [32]. The ‘metacount’ function was utilized to analyze data from mouse models, as all the included studies provided mean difference (MD) and 95% limits of confidence interval. On the other hand, the ‘metainc’ function was employed to analyze data from clinical patients, as all the studies reported the successful rate of OT. A significance level of *p* value < 0.05 was considered statistically significant for each main analysis. In cases where more than two subgroups were involved in a subgroup analysis, the *p* values were adjusted using the Bonferroni correction. This study adhered to the Preferred Reporting Items for Systematic Reviews and Meta-Analyses (PRISMA) guideline [33].

Higgins et al. [34] suggested that I^2^ values of 25%, 50%, and 75% stand for low, moderate, and high thresholds of heterogeneity, respectively. When conducting a meta-analysis, a low heterogeneity suggests minimal variability between studies that cannot be attributed to chance alone. Similarly, a meta-analysis with a low heterogeneity (25% ≤ I^2^ < 50%) indicates that the observed variability has only a small impact. Therefore, in this study, a common-effects framework was employed for studies without heterogeneity or with a low heterogeneity, while a random-effects framework was instead used for studies with a moderate or high heterogeneity.

### 2.6. Publication Bias and Heterogeneity Analysis

To assess publication bias, a sensitivity analysis was conducted by systematically excluding one study at a time using the ‘meta’ package [32]. Funnel plots were generated to visually examine potential publication bias, while Egger’s test, Begg’s test, and Thompson’s test were applied to quantify its extent [35]. *p* values greater than 0.1 were considered indicative of no publication bias.

Following the Cochrane System Intervention Review Manual recommendations, a subgroup analysis was conducted for outcome indicators to assess the reliability of the overall pooled analysis, and specific subgroup analyses were provided in cases where the number of included studies exceeded five. Meta-regression analysis was employed to explore potential sources of heterogeneity in cases of moderate or high heterogeneity in the meta-analysis. Test statistics and confidence intervals were adjusted using the Hartung and Knapp method [36]. Additionally, a radial plot was generated to estimate each study’s contribution to the overall heterogeneity.

## 3. Results

### 3.1. Summary of Included Studies

The PRISMA flowchart presents the main research process used for selecting eligible studies for the quantified meta-analysis (Figure 1). From an initial pool of 730 retrieved articles, a total of 16 studies were identified for a final quantitative synthesis. Among these, five studies investigated the effects of crustacean allergen on OIT in mouse models (Table 1), of which three studies focused on the major allergen from crab [28,37,38], and the other two studies examined the major allergen from shrimp [39,40]. Three of these studies evaluated the intervention efficacy of TM [38,39,40] on OIT, while the remaining two explored the efficacy of AK [28,37]. The processing methods varied across the studies, including Maillard reaction (MR) in two studies [37,38], enzymatic cross-linking approach in one study [28], epitope peptide in one study [39], and a low-dosage allergen treatment in one study [40].

Eleven studies were included in this study, investigating the intervention efficacy of slightly processed meat from fish and crustacea in clinical patients (Table 2). Notably, six of these studies only recruited one patient (<3) and assessed the efficacy of the major allergen from cod fish [8,41,42,43,44,45]. Due to the high consistency in the types of major allergens and fish among the six studies, they were combined into one overall study called Combination-6 for the quantitative analyses. In total, six clinical studies were included, with four utilizing processed meat from cod fish [46,47,48] and one each from salmon [49] and shrimp [50]. The processing methods in these studies included simple cooking in three studies [46,48,50], enzymatic hydrolysis of protease in one study [49], and lyophilization in one study [47]. The Combination-6 study employed multiple processing methods, including cooking alone [8,42], dehydration and cooking [41,43], lyophilization [44], and dilution [45]. Notably, the simple cooking method used in these studies typically involved boiling shrimp or fish meat at 100 °C or higher for 3 to 15 min to ensure thorough cooking. This consistency in the methods employed across these studies is also indicative of their homogeneity in processing methods.

As shown in Table 1, all the five studies in mouse models presented the anaphylactic score of mouse. Other indicators included the levels of multiple specific antibodies such as IgE, IgG1, and IgG2a, as well as cytokines like IL-4, IL-10, and IL-13. Two of the studies also reported the expression level of main effector cell populations, including eosinophils, mast cells, and goblet cells. A successful induction of OT in allergic mouse models was supported by reduced allergy symptoms, decreased expression levels of specific antibodies, and a reduced ratio of Th2/Th1. Regarding the six studies involving clinical patients, all of them reported the main incidence rate of OT challenge before and after OT treatment. Four of these studies also reported the levels of specific IgE antibody, and one study reported the wheal size as an indirect measurement of immunologic tolerance acquired after OIT treatment. Additionally, the presence or absence of allergic symptoms in patients helped to determine if they experienced allergic reactions.

### 3.2. Quality Assessment

With the widely used Newcastle–Ottawa quality assessment scale, we evaluated the accurate quality level for all the 16 included studies (Supplementary Table S1, ST1). Regarding the five case–control studies conducted with mouse models, all of them were deemed high-quality studies, receiving a score of 8 stars. These studies were considered of a high quality because they correctly chose the appropriate mouse models, clearly defined the cases and controls, selected the most important factor to study controls, had a secure record for exposure, and utilized the same method of ascertainment for cases and controls (ST1). In contrast, among the 11 cohort and case report studies involving clinical patients, most of them were rated as moderate-quality studies, receiving a score of 5 stars. This was because these studies did not demonstrate representativeness of the exposed cohort, select the non-exposed cohort, or select the most important factor to study controls (ST1). However, one study by Elbadawy et al. [46] was considered a high-quality study, receiving a score of 8 stars, as it met the requirements for selecting a non-exposed cohort and selecting the most important factor to study controls. Notably, the quality score was utilized to guide the interpretation of the pooled results rather than to exclude any study from quantitative analysis.

### 3.3. Risk of Publication Bias

To assess potential publication bias in the meta-analyses evaluating the intervention efficacy of the main indicator on OIT in mouse models and clinical patients, we conducted a publication bias analysis. For the five included studies in mouse models, the funnel plot, after being adjusted with the trim and filled method, appeared to be basically symmetrical (Supplementary Figure S1A). Additionally, all the three bias testing methods, including Egger’s test (*p* = 0.98), Begg’s test (*p* = 1.00), and Thompson’s test (*p* = 0.68), did not indicate any publication bias (*p* > 0.1). In contrast, the funnel plot and Egger’s test for the six included studies in clinical patients revealed a clear publication bias (*p* = 0.0006, Supplementary Figure S1B). In light of this, we conducted further sensitivity and meta-regression analyses to assess the stability of the pooled effects, as described below.

### 3.4. Main Results of Meta-Analysis

#### Intervention Efficacy of Crustacean Allergen in OIT in Mouse Model

**Figure 2:**
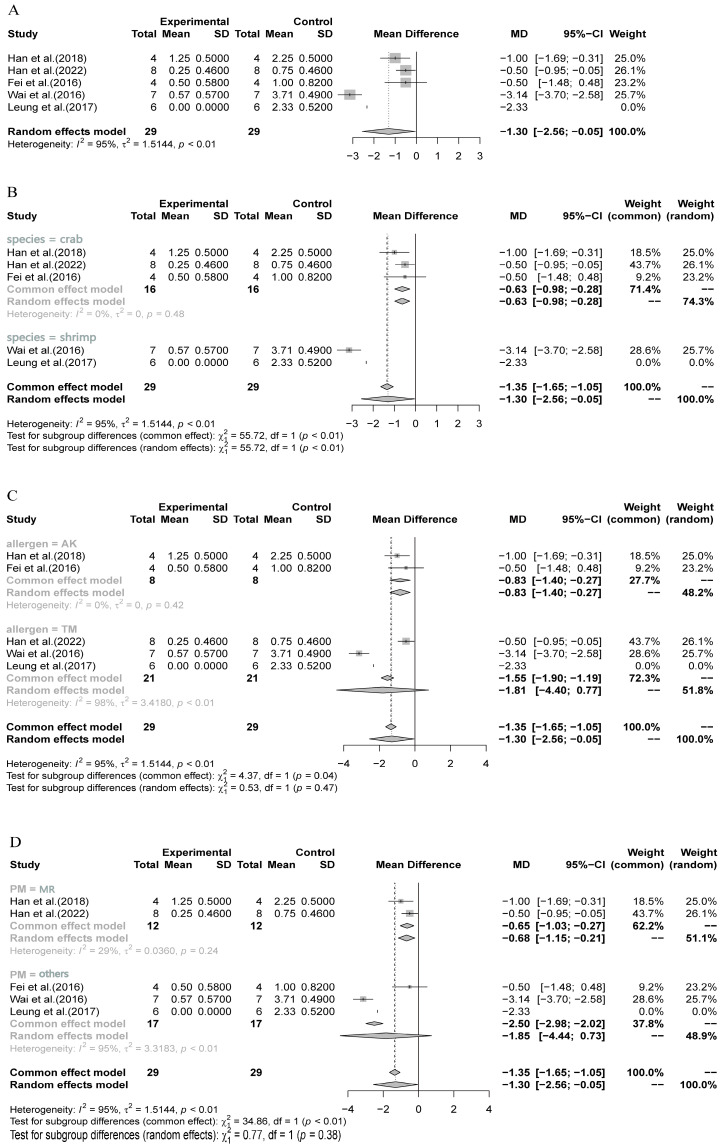
Main and subgroup meta-analyses of crustacean allergens in OIT in mouse models. (**A**) Main meta-analysis; (**B**) subgroup analysis of crustacean species (crab and shrimp); (**C**) subgroup analysis of allergen types (AK and TM); (**D**) subgroup analysis of different processing methods (MR and others). SD: standard deviation; MD: mean difference [28,37,38,39,40].

In our series of meta-analyses, we considered the anaphylactic score in mice as the main indicator, which was substantially supported by other measured indicators such as multiple specific antibodies, cytokines, and the expression level of main effector cell populations. We observed a high heterogeneity among these studies (I^2^ = 95%, *p* < 0.01) and, thus, employed the random-effects model to ensure the reliability of pooled results. The main meta-analysis found a substantial reduction in the anaphylactic score after OIT treatment (MD = −1.30, 95% CI: −2.56, −0.05; *p* < 0.01) (Figure 2A, Table 3), indicating a good efficacy of processed crustacean allergens in OIT for mice.

In addition to the main meta-analysis, three sets of subgroup analyses were further performed based on crustacean species (crab or shrimp), allergen types (AK or TM), and processing methods (MR or others), respectively. As shown in Figure 2B, the three included studies investigating crab allergen in OIT did not show any heterogeneity (I^2^ = 0) and indicated a high reliability of significant reduction for crab allergen in OIT (MD = −0.63, 95% CI: −0.98, −0.28) (Table 3). However, there was a very high heterogeneity between the crab and shrimp subgroups (I^2^ = 95%), which was also supported by the test for subgroup differences (*p* < 0.01). Regarding the subgroups of AK and TM, the two studies for AK did not show any heterogeneity (I^2^ = 0) and indicated a substantial reduction in anaphylactic score in this subgroup (MD = −0.83, 95% CI: −1.40, −0.27) (Figure 2C, Table 3). In contrast, the three studies for TM allergen showed a very high level of heterogeneity (I^2^ = 98%). This suggests the need for a random-effects model for the meta-analysis, which did not yield a significant outcome (MD = −1.81, 95% CI: −4.40, 0.77) (Figure 2C). For the subgroup analysis of processing methods, the two included studies for MR showed a low level of heterogeneity (I^2^ = 29%) and demonstrated a significant reduction in the anaphylactic score (MD = −0.65, 95% CI: −1.03, −0.27, common-effects model) (Figure 2D, Table 3). On the other hand, the three studies for other methods (enzymatic cross-linking, epitope peptide, and low-dosage methods) accounted for most of the overall heterogeneity (I^2^ = 95%) and did not yield a significant pooled outcome (MD = −1.85, 95% CI: −4.44, 0.73, random-effects model) (Figure 2D).

### 3.5. Intervention Efficacy of Processed Meat from Fish and Crustacea in OIT in Clinical Patients

**Figure 3:**
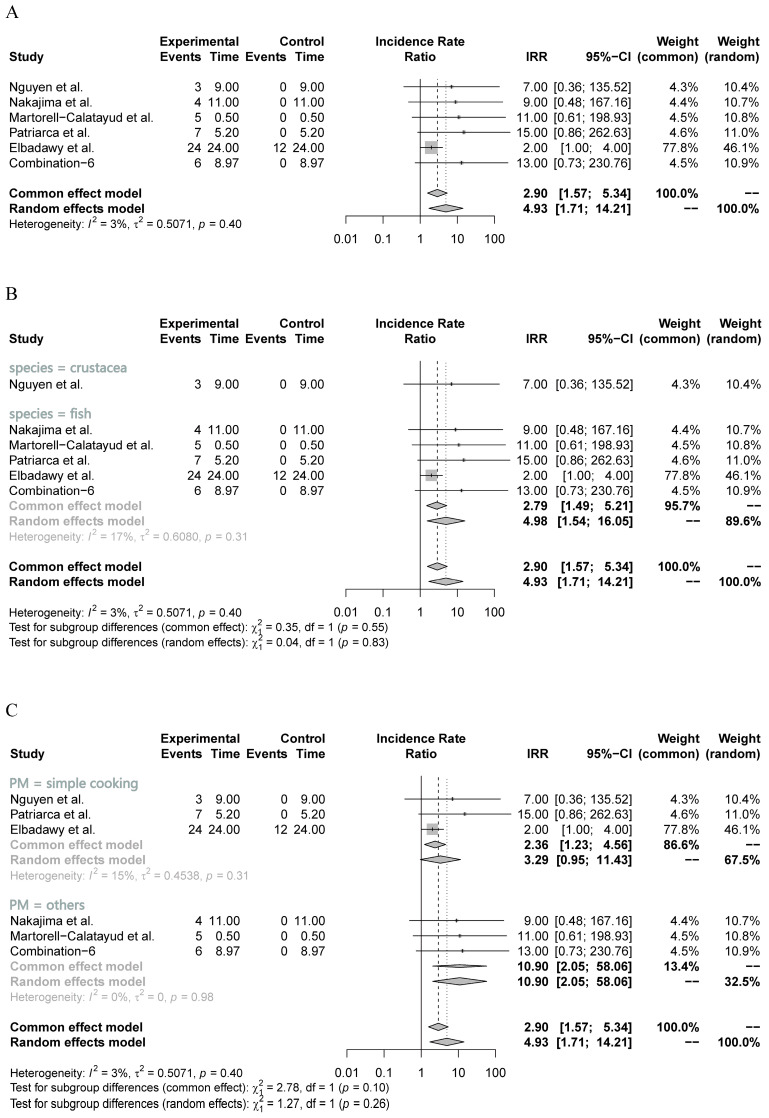
Main and subgroup meta-analyses of seafood meat in OIT in clinical patients. (**A**) Main meta-analysis; (**B**) subgroup analysis of seafood species (crustacea and fish); (**C**) subgroup analysis of different processing methods (simple cooking and others). IRR: incidence rate ratio [46,47,48,49,50].

To assess the intervention efficacy in clinical patients, the main indicator used was the incidence rate of OT. This incidence rate was supported by the level of specific IgE antibody and the wheal size in the six included studies. In the main meta-analysis, a very low heterogeneity (I^2^ = 3%) was observed among the included studies, leading to the adoption of the common-effects model. As depicted in Figure 3A, the main meta-analysis revealed a substantial increase in the OT incidence rate following OIT treatment (IRR = 2.90, 95% CI: 1.57, 5.34, *p* < 0.01) (Table 3). These findings suggest that clinical patients substantially improve their ability to develop OT after undergoing OIT treatment, despite individual studies not reporting any significant IRR.

Furthermore, two sets of subgroup analyses were conducted based on seafood species (fish or crustacea) and processing methods (cooking or others). As shown in Figure 3B, the five included studies evaluating the efficacy of processed fish meat in OIT showed a relatively low heterogeneity (I^2^ = 17%), indicating the relatively high reliability of the pooled results. The subgroup analysis demonstrated a significant increase in the OT incidence rate when utilizing processed fish meat as the resource for OIT (IRR = 2.79, 95% CI: 1.49, 5.21, *p* < 0.01) (Figure 3B). Although the subgroup analysis of processed crustacean meat also generated a significant outcome, it only included one small study (*n* = 3), which remarkably reduced the reliability of pooled results. Regarding the subgroup analysis of processing methods, the three included studies that investigated the simple cooking approach exhibited a low heterogeneity (I^2^ = 15%) and identified a substantial increase in the OT incidence rate (IRR = 2.36, 95% CI: 1.23, 4.56, *p* = 0.01) (Figure 3C). However, the remaining three studies did not provide sufficient evidence to establish a reliable outcome for one specific method, despite the pooled results of other methods indicating a significant increase (Figure 3C).

### 3.6. Sensitivity and Meta-Regression Analyses

**Figure 4:**
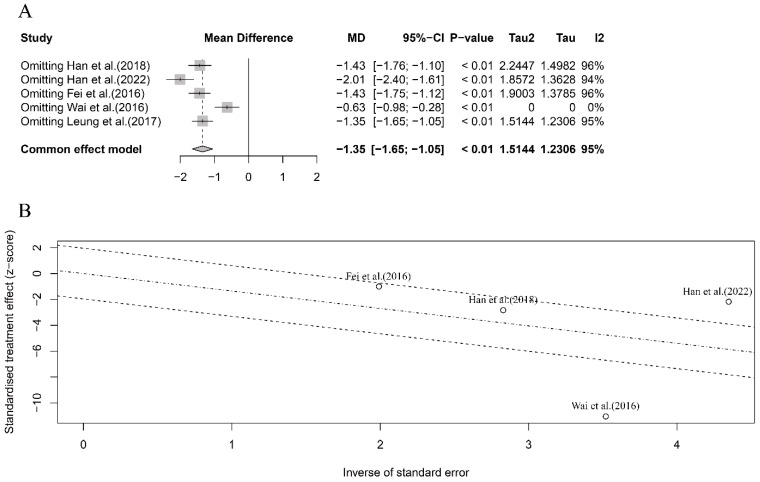
Sensitivity analysis (**A**) and meta-regression analysis (**B**) for the intervention efficacy of crustacean allergens in OIT in mouse model. Note: Leung et al. (2017)’s study was excluded from heterogeneity analysis as the standard deviation of anaphylactic score after OIT treatment was zero [28,37,38,39,40].

In this part, we conducted sensitivity and meta-regression analyses to identify any specific study that had a substantial contribution to the overall heterogeneity. For the five case–control studies in the mouse model, the sensitivity analysis revealed that the study by Wai et al. [39] had a significant influence on the pooled results (Figure 4A). The overall heterogeneity was reduced to zero when excluding this study, indicating that it accounted for most of the overall heterogeneity (Figure 4A,B). However, excluding Wai et al.’s study [39] did not change the significant pooled results (MD = −0.63, 95% CI: −0.98, −0.28, *p* < 0.01) (Figure 4A). Furthermore, the meta-regression analysis identified the crustacean species (crab or shrimp) as the significant source contributing to the overall heterogeneity (*p* = 0.013).

**Figure 5:**
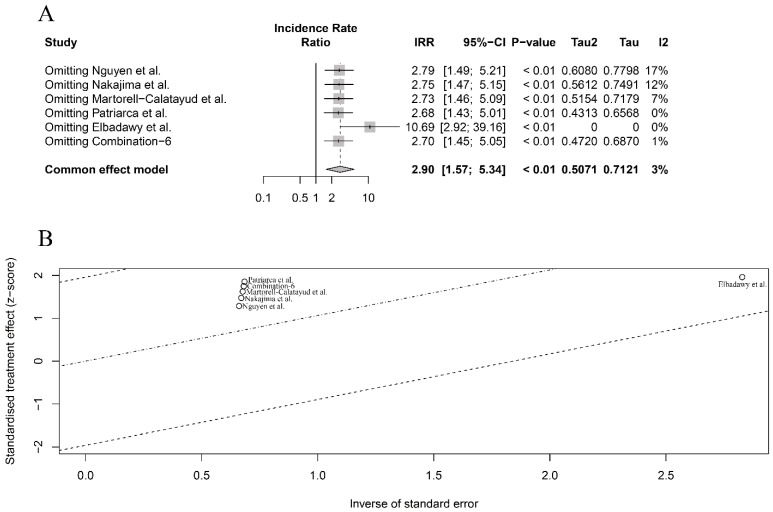
Sensitivity analysis (**A**) and meta-regression analysis (**B**) for intervention efficacy of seafood meat in OIT in clinical patients [46,47,48,49,50].

In terms of OIT efficacy in clinical patients, we also conducted sensitivity and meta-regression analyses for the six included studies, despite the low-level heterogeneity observed in the seafood species and processing methods subgroups, respectively (Figure 3B,C). The meta-regression analysis did not show any significant heterogeneity based on these two different subgroups (*p* = 0.90 and *p* = 0.72, respectively), which further confirmed the low-level overall heterogeneity in the six included studies. However, the sensitivity analysis identified that the study by Elbadawy et al. [46] had the largest influence on pooled results, although excluded, this study did not change the direction and significance of the pooled results (IRR > 1 and *p* < 0.01) (Figure 5A,B). Compared with the lower one in the overall meta-analysis (Figure 3A), the substantial influence of Elbadawy et al.’s study [46] on heterogeneity partially explained the relatively higher heterogeneity in the fish meat subgroup (Figure 3B) and cooking approach subgroup (Figure 3C).

## 4. Discussion

The present meta-analyses provided preliminary evidence that slightly processed crustacean allergens could significantly reduce the anaphylactic score in mice after OIT treatment, indicating a high intervention efficacy of processed crustacean allergens in the mouse models. Furthermore, more reliable pooled results were obtained in the three subgroups analyses based on crustacean species (crab), allergen types (AK), and processing methods (MR), respectively. Additionally, the present meta-analyses showed that slightly processed meat from fish and crustacea had a good efficacy in OIT in clinical patients, as it significantly increased the OT incidence rates for patients. The pooled results, with a very low heterogeneity, were considered highly reliable. Moreover, credible results were also observed in the subgroups of processed fish meat and a simple cooking approach, respectively.

### 4.1. Intervention Efficacy of Various Processing Methods for Allergens from Fish and Crustacea in OIT

The five included studies in the mouse models utilized multiple processing methods for crustacean allergens and showed a reliable pooled outcome in the subgroup analysis of the MR approach. This approach was found to significantly reduce allergic reactions in the mouse model and may hold a strong potential as a promising slightly processing method for clinical patients. Currently, clinicians and scientists commonly utilize the simple cooking method for seafood meat in clinical patients. Although we obtained positive pooled results for this strategy (IRR = 2.36, *p* = 0.01), the included studies were affected by an obvious publication bias. In general, the cooking method is believed to change or destroy conformational epitopes in food proteins, thereby reducing the intensity of allergic reactions while maintaining the potential for OT [12]. However, the widespread adoption of this simple method is hindered by the relatively high rate of serious adverse reactions and the uncertainty surrounding variations during the treatment process [19,51].

The MR approach has gained popularity over the simple cooking method due to the relatively clear reactions when different types of natural sugars are added during the heating process [29]. This reaction is simple and does not require any special chemical materials or sophisticated equipment. Meanwhile, the mechanism underlying the changes in allergenicity with MR is clear, which is primarily through the destruction of conformational epitopes, exposure of hidden epitopes, and masking of linear epitopes [52]. However, the changes in allergenicity are known to be dependent on the source of the allergen and the specific allergen itself. Most types of sugars for glycating shrimp TM could significantly reduce allergen’s IgE reactivity *in vitro* and *in vivo*, except the maltose that may be due to the formation of AGEs as neoallergens [30]. Another study observed that among five types of sugars, only arabinose was effective in reducing AK’s IgE-binding capacity and decreasing its allergenicity *in vivo* [37]. For the large families of fish and shellfish, the MR approach should be carefully controlled by considering the specific types of sugar to avoid the generation of neoallergens [53,54]. Moreover, the OT potential of MR-processed allergen should be further assessed *in vivo* because the processed allergen may not only reduce its allergenicity *in vitro*, but might also attenuate its ability to stimulate the OT potential *in vivo* [28].

### 4.2. Intervention Efficacy and Safety of Different Allergens in OIT in Clinical Patients

Currently, most OIT clinical trials have focused on three types of allergens from cow milk, hen eggs, and peanuts, which have shown good progress for clinical patients [55,56,57]. However, there are only a few standard randomized control trials (RCTs) that comprehensively evaluated the efficacy of seafood allergen/meat in OIT for clinical patients, and the efficacy remains controversial. Our meta-analysis included six studies in clinical patients and identified a good efficacy of slightly processed seafood meat in OIT, with no reports of severe allergic reactions. This indicates a relatively high potential for the MR and simple cooking treatments of seafood allergens in future well-designed RCTs. In addition to the intervention efficacy, the safety of an allergen and a patient’s tolerance are also important evaluation indicators for assessing the potential application of processed allergens in clinical allergic patients. It has been reported that most severe reactions occur during the initial rapid dose escalation phase and the build-up phase of the desensitization process [58]. The rate of severe allergic reactions is significantly associated with various confounding factors, such as a loss of asthma control, treatment during limosis, excessive physical exertion after intervention, and other comorbidities [59]. Therefore, the intervention strategy should comprehensively control these factors to improve safety and reduce the occurrence rate of severe allergic reactions. Furthermore, a combined intervention strategy of processed seafood allergen with omalizumab antibody or food allergy herbal formula 2 may significantly reduce the rate of severe allergic reactions while increasing the successful rate of desensitization [10,60]. Although mild reactions were reported in several of the included studies in this study, it is important to note that the majority of patients experienced self-recovery without requiring medical intervention. These findings provide useful advice for designing standard RCTs of slightly processed seafood allergen/meat in OIT in clinical patients.

## 5. Limitations

There were three main limitations in the present study. Firstly, there was a high level of heterogeneity in the main meta-analysis of crustacean allergen in OIT in the mouse models, which would potentially impact the reliability of the pooled results. However, the heterogeneity decreased to a very low level in the MR subgroup and was zero in the crab species and AK allergen subgroups. This supports the highly reliable pooled results in these three subgroups. Additionally, we identified the study by Wai et al. [39] as the main source contributing to the overall heterogeneity and confirmed that the exclusion of this study did not change the direction and significance of the pooled results. Secondly, the six OIT studies in clinical patients included a small number of cases and tended to report successful cases, which may contribute to a potential publication bias. Moreover, most of the six clinical studies, except for the study by Elbadawy et al. [46], directly evaluated the efficacy of processed meat from fish and crustacea in OIT in the recruited cases but did not include a placebo group. This could reduce the reliability of the pooled results. Fortunately, the six included studies only generated a very low heterogeneity in both the main and subgroup analyses, ensuring the high reliability of the pooled results. Finally, it is important to note that this study did not include research specifically investigating the efficacy of OIT using processed allergens from molluscan shellfish, despite the fact that the defined search terms covered this species. Therefore, it is worthwhile to conduct further research and stay updated on the latest findings in this area to enhance and expand upon the findings of this study.

## 6. Conclusions

In conclusion, the present meta-analysis demonstrated that the slightly processed crustacean allergens may have the ability to significantly reduce allergic reactions without inducing severe allergic reactions in the mouse model. Specifically, the crab species, AK allergen, and MR processing method showed promising results in inducing OT. Furthermore, the simple processing method of cooking also showed success in inducing OT potential in clinical patients. The high incidence rate of OT and the relatively low rate of severe adverse effects make the OIT intervention treatment a promising and specific approach to treating seafood allergy. However, these findings should be interpreted with caution and need further support from large RCTs to confirm the efficacy and safety of these processing approaches.

## Figures and Tables

**Table 1:** Included case–control studies for exploring intervention efficacy of processed crustacean allergens on OIT in mouse models.

First Author, Year (Reference)	Design	Participants	Processing Methods ^a^	Utilized Allergen ^b^	Main Indicators	Other Indicators
Han et al. (2018) [37]	Case– control	8 mice in allergic model and equal controls	MR	Crab, AK	Anaphylactic score	Multiple specific antibodies and cytokines
Han et al. (2022) [38]	Case– control	4 mice in allergic model and equal controls	MR	Crab, TM	Anaphylactic score	Multiple specific antibodies and cytokines
Fei et al. (2016) [28]	Case– control	4 mice in allergic model and equal controls	Enzymatic cross-linking	Crab, AK	Anaphylactic score	Multiple specific antibodies and cytokines
Wai et al. (2016) [39]	Case– control	7 mice in allergic model and equal controls	Epitope peptide	Shrimp, TM	Anaphylactic score	Multiple specific antibodies and cytokines, main effector cell clusters
Leung et al. (2017) [40]	Case– control	6 mice in allergic model and equal controls	Low-dosage	Shrimp, TM	Anaphylactic score	Multiple specific antibodies and cytokines, main effector cell clusters

^a^ MR: Maillard reaction; ^b^ TM: tropomyosin; AK: arginine kinase.

**Table 2:** Included cohort studies investigating intervention efficacy of processed seafood meat in OIT in clinical patients.

First Author, Year (Reference)	Design	Age	Participants	Processing Methods	Utilized Meat	Main Indicators	Other Indicators ^b^
Nguyen et al. (2022) [50]	Cohort	5–21	2 male and 1 female	Cooking	Shrimp, meat	OT rate	OT dosage; overall and specific IgE; wheal size
Ugajin et al. (2021) [8]	Case report	20	1 female	Cooking	Cod fish, meat	OT rate	OT dosage; BAT; IgE; allergic symptoms
Porcaro et al. (2016) [41]	Case report	11	1 male	Dehydration and cooking	Cod fish, meat	OT rate	OT dosage; IgE; wheal size
Nucera et al. (2018) [42]	Case report	20	1 male	Cooking	Cod fish, meat	OT rate	OT dosage
Nakajima et al. (2015) [49]	Cohort	ND ^a^	ND	Enzymatic hydrolysis of protease	Salmon, meat	OT rate	OT dosage; allergic symptoms
Damelio et al. (2015) [43]	Case report	6	1 female	Dehydration and cooking	Cod fish, meat	OT rate	OT dosage; IgE; wheal size
Elbadawy et al. (2017) [46]	Cohort	Child	36 cases	Cooking	Cod fish, meat	Yes (66.7%) No (33.3%)	OT dosage
D’Amelio et al. (2016) [44]	Case report	6	1 female	Lyophilization	Cod fish, meat	OT rate	OT dosage; wheal size
Casimir et al. (1997) [45]	Case report	3.25	1 female	Dilution	Cod fish, meat	OT rate	OT dosage; allergic symptoms
Martorell-Calatayud et al. (2019) [47]	Cohort	4–14	5 cases	Lyophilization	Cod fish, meat	OT rate	OT dosage; allergic symptoms
Patriarca et al. (2007) [48]	Cohort	5–15	7 cases	Cooking	Cod fish, meat	OT rate	OT dosage; allergic symptoms

^a^ ND: no data; ^b^ OT: oral tolerance.

**Table 3:** Pooled results of intervention efficacy of processed allergen/meat from fish and crustacea in OIT in mouse models and clinical patients, respectively.

Outcome Type and Indicators ^a^	Studies, *n*	*p*	Significance of Threshold ^c^	MD/IRR (95% CI) ^d^	I^2^, %
**Mouse models—overall**	5	<0.01	0.05	−1.30 (−2.56, −0.05)	95
**Three subgroups**					
Species—crab	3	<0.01	0.025	−0.63 (−0.98, −0.28)	0
Species—shrimp	2 ^b^	--	0.025	--	--
Allergen—AK	2	<0.01	0.025	−0.83 (−1.40, −0.27)	0
Allergen—TM	3	0.17	0.025	−1.81 (−4.40, 0.77)	98
PM-MR	2	<0.01	0.025	−0.65 (−1.03, −0.27)	29
PM—others	3	0.16	0.025	−1.85 (−4.44, 0.73)	95
**Clinical patients—overall**	6	<0.01	0.05	2.90 (1.57, 5.34)	3
**Two subgroups**					
Species—crustacea	1	--	0.025	7.00 (0.36, 135.52)	--
Species—fish	5	<0.01	0.025	2.79 (1.49, 5.21)	17
PM—simple cooking	3	0.01	0.025	2.36 (1.23, 4.56)	15
PM—others	3	<0.01	0.025	10.90 (2.05, 58.06)	0

^a^ AK: arginine kinase; TM: tropomyosin; PM: processing method; MR: Maillard reaction. ^b^ The two studies cannot be combined because the standard deviation of the anaphylactic score after OIT treatment in Leung et al.’s study is zero [40]. ^c^ Significance of threshold: Significance threshold after Bonferroni correction. ^d^ The MD (mean difference) statistic was applied to mouse models. The IRR (incidence rate ratio) was applied to clinical patients. --: data are not available.

## Data Availability

The original contributions presented in the study are included in the article/Supplementary Materials, further inquiries can be directed to the corresponding author/s.

## Supplementary Materials

The following supporting information can be downloaded at: https://www.mdpi.com/article/10.3390/nu16050667/s1, Table S1: Quality assessments for 5 case–control studies in mouse models and 11 cohort/case report studies in clinical patients; Figure S1: Funnel plots for evaluating publication bias for intervention efficacy of seafood allergen in OIT. (A) Trim and filled methods for included studies in mouse models; (B) Included studies in clinical patients.

## References

[1] Sicherer S.H., Sampson H.A. (2018). Food allergy: A review and update on epidemiology, pathogenesis, diagnosis, prevention, and management. J. Allergy Clin. Immunol..

[2] Berin M.C. (2019). Mechanisms that define transient versus persistent food allergy. J. Allergy Clin. Immunol..

[3] Baumert J., Brooke-Taylor S., Chen H., Crevel R.W., Houben G.F., Jackson L., Kyriakidis S., La Vieille S., Lee N.A., López M.C. (2021). Part 1: Review and Validation of Codex Priority Allergen List through Risk Assessment.

[4] Kanny G., Dano D., Danan J.-L., Astier C., Lefevre S. (2015). Information des consommateurs allergiques et étiquetage: Actualités. Rev. Fr. D’allergologie.

[5] Wai C.Y.Y., Leung N.Y.H., Leung A.S.Y., Wong G.W.K., Leung T.F. (2021). Seafood Allergy in Asia: Geographical Specificity and Beyond. Front. Allergy.

[6] Skypala I. (2011). Adverse food reactions--an emerging issue for adults. J. Am. Diet. Assoc..

[7] Davis C.M., Gupta R.S., Aktas O.N., Diaz V., Kamath S.D., Lopata A.L. (2020). Clinical Management of Seafood Allergy. J. Allergy Clin. Immunol. Pract..

[8] Ugajin T., Kobayashi Y., Takayama K., Yokozeki H. (2021). A parvalbumin allergy case was successfully treated with oral immunotherapy using hypoallergenic fish. Allergol. Int..

[9] Sampson H.A., Leung D.Y., Burks A.W., Lack G., Bahna S.L., Jones S.M., Wong D.A. (2011). A phase II, randomized, doubleblind, parallelgroup, placebocontrolled oral food challenge trial of Xolair (omalizumab) in peanut allergy. J. Allergy Clin. Immunol..

[10] Brandström J., Vetander M., Sundqvist A.C., Lilja G., Johansson S.G.O., Melén E., Sverremark-Ekström E., Nopp A., Nilsson C. (2019). Individually dosed omalizumab facilitates peanut oral immunotherapy in peanut allergic adolescents. Clin. Exp. Allergy.

[11] Nowak-Wegrzyn A., Szajewska H., Lack G. (2017). Food allergy and the gut. Nat. Rev. Gastroenterol. Hepatol..

[12] Yu W., Freeland D.M.H., Nadeau K.C. (2016). Food allergy: Immune mechanisms, diagnosis and immunotherapy. Nat. Rev. Immunol..

[13] Wood R.A. (2017). Oral immunotherapy for food allergy. J. Investig. Allergol. Clin. Immunol..

[14] Ryan J.F., Hovde R., Glanville J., Lyu S.C., Ji X., Gupta S., Tibshirani R.J., Jay D.C., Boyd S.D., Chinthrajah R.S. (2016). Successful immunotherapy induces previously unidentified allergen-specific CD4+ T-cell subsets. Proc. Natl. Acad. Sci. USA.

[15] Dougherty J.A., Wagner J.D., Stanton M.C. (2021). Peanut Allergen Powder-dnfp: A Novel Oral Immunotherapy to Mitigate Peanut Allergy. Ann. Pharmacother..

[16] Wongteerayanee C., Tanticharoenwiwat P., Rutrakool N., Senavonge A., Jeekungwal N., Pacharn P., Vichyanond P. (2020). Feasibility of a 3-step protocol of wheat oral immunotherapy in children with severe wheat allergy. Asia Pac. Allergy.

[17] Palosuo K., Karisola P., Savinko T., Fyhrquist N., Alenius H., Mäkelä M.J., Randomized A. (2021). Open-Label Trial of Hen’s Egg Oral Immunotherapy: Efficacy and Humoral Immune Responses in 50 Children. J. Allergy Clin. Immunol. Pract..

[18] Vickery B.P., Vereda A., Casale T.B., Beyer K., Toit G.D., Hourihane J.O., Jones S.M., Shreffler W.G., Marcantonio A., Zawadzki R. (2018). AR101 Oral Immunotherapy for Peanut Allergy. N. Engl. J. Med..

[19] Larsen J.M., Bang-Berthelsen C.H., Qvortrup K., Sancho A.I., Hansen A.H., Andersen K.I.H., Thacker S.S.N., Eiwegger T., Upton J., Bøgh K.L. (2020). Production of allergen-specific immunotherapeutic agents for the treatment of food allergy. Crit. Rev. Biotechnol..

[20] Bai T.L., Han X.Y., Li M.S., Yang Y., Liu M., Ji N.R., Yu C.C., Lai D., Cao M.J., Liu G.M. (2021). Effects of the Maillard reaction on the epitopes and immunoreactivity of tropomyosin, a major allergen in Chlamys nobilis. Food Funct..

[21] Ahmed I., Lin H., Xu L., Li S., Costa J., Mafra I., Chen G., Gao X., Li Z. (2020). Immunomodulatory Effect of Laccase/Caffeic Acid and Transglutaminase in Alleviating Shrimp Tropomyosin (Met e 1) Allergenicity. J. Agric. Food Chem..

[22] Fu L., Ni S., Wang C., Wang Y. (2019). Transglutaminase-catalysed cross-linking eliminates Penaeus chinensis tropomyosin allergenicity by altering protein structure. Food Agric. Immunol..

[23] Shimakura K., Tonomura Y., Hamada Y., Nagashima Y., Shiomi K. (2005). Allergenicity of crustacean extractives and its reduction by protease digestion. Food Chem..

[24] Saifi M., Clark A., Arneson A., Feldman M., Bird J.A. (2015). Baked egg oral immunotherapy (OIT) for baked egg (BE) allergic children. J. Allergy Clin. Immunol..

[25] Amat F., Bourgoin-Heck M., Lambert N., Deschildre A., Just J. (2017). Immunothérapie orale au lait: Cru ou cuit?. Rev. Française D’allergologie.

[26] Mattar H., Padfield P., Simpson A., Mills E.N.C. (2021). The impact of a baked muffin matrix on the bioaccessibility and IgE reactivity of egg and peanut allergens. Food Chem..

[27] Liu G., Hu M., Sun L.-C., Han X., Liu Q., Alcocer M., Fei D., Cao M.-J., Liu G.-M. (2017). Allergenicity and oral tolerance of enzymatic cross-linked tropomyosin evaluated using cell and mouse models. J. Agric. Food Chem..

[28] Fei D.X., Liu Q.M., Chen F., Yang Y., Chen Z.W., Cao M.J., Liu G.M. (2016). Assessment of the sensitizing capacity and allergenicity of enzymatic cross-linked arginine kinase, the crab allergen. Mol. Nutr. Food Res..

[29] Gou J., Liang R., Huang H., Ma X. (2022). Maillard Reaction Induced Changes in Allergenicity of Food. Foods.

[30] Zhang Z., Xiao H., Zhou P. (2019). Allergenicity suppression of tropomyosin from Exopalaemon modestus by glycation with saccharides of different molecular sizes. Food Chem..

[31] Nakamura A., Watanabe K., Ojima T., Ahn D.H., Saeki H. (2005). Effect of maillard reaction on allergenicity of scallop tropomyosin. J. Agric. Food Chem..

[32] Balduzzi S., Rücker G., Schwarzer G. (2019). How to perform a meta-analysis with R: A practical tutorial. Evid. Based Ment. Health.

[33] Page M.J., McKenzie J.E., Bossuyt P.M., Boutron I., Hoffmann T.C., Mulrow C.D., Shamseer L., Tetzlaff J.M., Akl E.A., Brennan S.E. (2021). 2020 statement: An updated guideline for reporting systematic reviews. BMJ.

[34] Higgins J.P., Thompson S.G., Deeks J.J., Altman D.G. (2003). Measuring inconsistency in meta-analyses. BMJ.

[35] Irwig L., Macaskill P., Berry G., Glasziou P. (1998). Bias in meta-analysis detected by a simple, graphical test. Graphical test is itself biased. BMJ Br. Med. J..

[36] Viechtbauer W. (2010). Conducting meta-analyses in R with the metafor package. J. Stat. Softw..

[37] Han X.Y., Yang H., Rao S.T., Liu G.Y., Hu M.J., Zeng B.C., Cao M.J., Liu G.M. (2018). The Maillard Reaction Reduced the Sensitization of Tropomyosin and Arginine Kinase from Scylla paramamosain, Simultaneously. J. Agric. Food Chem..

[38] Han X.-Y., Bai T.-L., Yang H., Lin Y.-C., Ji N.-R., Wang Y.-B., Fu L.-L., Cao M.-J., Liu J.-W., Liu G.-M. (2022). Reduction in Allergenicity and Induction of Oral Tolerance of Glycated Tropomyosin from Crab. Molecules.

[39] Wai C., Leung N., Leung P., Chu K. (2016). T cell epitope immunotherapy ameliorates allergic responses in a murine model of shrimp allergy. Clin. Exp. Allergy.

[40] Leung N.Y.H., Wai C.Y.Y., Shu S.A., Chang C.C., Chu K.H., Leung P.S. (2017). Low-dose allergen-specific immunotherapy induces tolerance in a murine model of shrimp allergy. Int. Arch. Allergy Immunol..

[41] Porcaro F., Caminiti L., Crisafulli G., Arasi S., Chiera F., La Monica G., Pajno G.B. (2016). Management of Food Allergy to Fish with Oral Immunotherapy: A Pediatric Case Report. Pediatr. Allergy Immunol. Pulmonol..

[42] Nucera E., Ricci A.G., Rizzi A., Mezzacappa S., Di Rienzo A., Pecora V., Patriarca G., Buonomo A., Aruanno A., Schiavino D. (2018). Specific oral immunotherapy in food allergic patients: Transient or persistent tolerance?. Adv. Dermatol. Allergol. Postępy Dermatol. I Alergol..

[43] Damelio C.M. (2015). Successful Specific Oral Tolerance Induction with Hake in an Allergic Child Detecting Fish in Cooking Steam. J. Allergy Clin. Immunol..

[44] D’Amelio C., Gastaminza G., Vega O., Bernad A., Madamba R., Martínez-Aranguren R., Ferrer M., Goikoetxea M. (2016). Induction of tolerance to different types of fish through desensitization with hake. Pediatr. Allergy Immunol. Off. Publ. Eur. Soc. Pediatr. Allergy Immunol..

[45] Casimir G., Cuvelier P., AIIard S., Duchateau J. (1997). Life-threatening fish allergy successfully treated with immunotherapy. Pediatr. Allergy Immunol..

[46] ELBadawy N.E., Abdel-Latif R.S. (2017). Food specific IgE as a biomarker of oral immunotherapy efficacy in comparison to double blind food challenge test. Egypt J. Immunol..

[47] Martorell-Calatayud C., Carnés J., Gómez A.M., Fernández L.D., Zudaire L.E., Rodríguez C.S., Galán C.G., Toral Pérez T., Rodríguez Del Río P., Martorell Aragonés A. (2019). Oral Immunotherapy to Hake in 8 Pediatric Patients. J. Investig. Allergol. Clin. Immunol..

[48] Patriarca G., Nucera E., Pollastrini E., Roncallo C., Pasquale T.D., Lombardo C., Pedone C., Gasbarrini G., Buonomo A., Schiavino D. (2007). Oral specific desensitization in food-allergic children. Dig. Dis. Sci..

[49] Nakajima Y., Kondo Y., Mori Y., Otaka S., Okubo Y., Tanaka K., Yamawaki K., Inuo C., Hirata N., Suzuki S. (2015). Oral Immunotherapy for Fish Allergy Using a Hypoallergenic Decomposed Fish Meat. J. Allergy Clin. Immunol..

[50] Nguyen D.-T.I., Sindher S.B., Chinthrajah R.S., Nadeau K., Davis C.M. (2022). Shrimp-allergic patients in a multi-food oral immunotherapy trial. Pediatr. Allergy Immunol..

[51] Yee C.S., Rachid R. (2016). The Heterogeneity of Oral Immunotherapy Clinical Trials: Implications and Future Directions. Curr. Allergy Asthma Rep..

[52] Toda M., Hellwig M., Henle T., Vieths S. (2019). Influence of the Maillard Reaction on the Allergenicity of Food Proteins and the Development of Allergic Inflammation. Curr. Allergy Asthma Rep..

[53] Zhang Z., Xiao H., Zhou P. (2019). Glycation by saccharides of different molecular sizes affected the allergenicity of shrimp tropomyosin via epitope loss and the generation of advanced glycation end products. Food Funct..

[54] Zhang Z., Li X.M., Xiao H., Nowak-Wegrzyn A., Zhou P. (2020). Insight into the allergenicity of shrimp tropomyosin glycated by functional oligosaccharides containing advanced glycation end products. Food Chem..

[55] Calatayud C.M., García A.M.A. (2014). Martorell Aragonés and B. De La Hoz Caballer, Safety and efficacy profile and immunological changes associated with oral immunotherapy for IgE-mediated cow’s milk allergy in children: Systematic review and meta-analysis. J. Investig. Allergol. Clin. Immunol..

[56] Kim E.H., Perry T.T., Wood R.A., Leung D.Y.M., Berin M.C., Burks A.W., Cho C.B., Jones S.M., Scurlock A., Sicherer S.H. (2020). Induction of sustained unresponsiveness after egg oral immunotherapy compared to baked egg therapy in children with egg allergy. J. Allergy Clin. Immunol..

[57] Sampson H.A. (2013). Peanut oral immunotherapy: Is it ready for clinical practice?. J. Allergy Clin. Immunol. Pract..

[58] Eapen A.A., Lavery W.J., Siddiqui J.S., Lierl M.B. (2019). Oral immunotherapy for multiple foods in a pediatric allergy clinic setting. Ann. Allergy Asthma Immunol..

[59] Varshney P., Steele P.H., Vickery B.P., Bird J.A., Thyagarajan A., Scurlock A.M., Perry T.T., Jones S.M., Burks A.W. (2009). Adverse reactions during peanut oral immunotherapy home dosing. J. Allergy Clin. Immunol..

[60] Srivastava K.D., Song Y., Yang N., Liu C., Goldberg I.E., Nowak-Węgrzyn A., Sampson H.A., Li X.M. (2017). B-FAHF-2 plus oral immunotherapy (OIT) is safer and more effective than OIT alone in a murine model of concurrent peanut/tree nut allergy. Clin. Exp. Allergy.

