# Supplementary material for: Intervention Efficacy of Slightly Processed Allergen/Meat in Oral Immunotherapy for Seafood Allergy: A Systematic Review, Meta-Analysis, and Meta-Regression Analysis in Mouse Models and Clinical Patients
Source: Nutrients. 2024 Feb 27;16(5):667. doi: 10.3390/nu16050667 (PMC10934674; doi:10.3390/nu16050667)
Supplement: Supplementary file 1 [file nutrients-16-00667-s001.zip › 3.supplementary.tables.figures-20240220.pdf]

Supplementary Table S1. Quality assessments for five case-control studies in mouse models and 11 cohort/case report studies in clinical patients.

Study	Selection	Comparability	Exposure/ Outcome [a]	Total score	Quality level
5 studies in mouse model					
Han et al.(2018)	4	2	2	8	High
Han et al.(2022)	4	2	2	8	High
Fei et al.(2016)	4	2	2	8	High
Wai et al.(2016)	4	2	2	8	High
Leung et al.(2017)	4	2	2	8	High
11 studies in clinical patients					
Nguyen et al.(2022)	2	0	3	5	Moderate
Ugajin et al.(2021)	2	0	3	5	Moderate
Porcaro et al.(2016)	2	0	3	5	Moderate
Nucera et al.(2018)	2	0	3	5	Moderate
Nakajima et al.(2015)	2	0	3	5	Moderate
Damelio et al.(2015)	2	0	3	5	Moderate
Elbadawy et al.(2017)	3	2	3	8	High
D'Amelio et al.(2017)	2	0	3	5	Moderate
Casimir et al.(1997)	2	0	3	5	Moderate
Martorell-Calatayud et al. (2019)	2	0	3	5	Moderate
Patriarca et al.(2007)	2	0	3	5	Moderate

[a] Exposure/outcome: Three items in exposure were applied in mouse models; three items in outcome were applied in clinical patients.

A

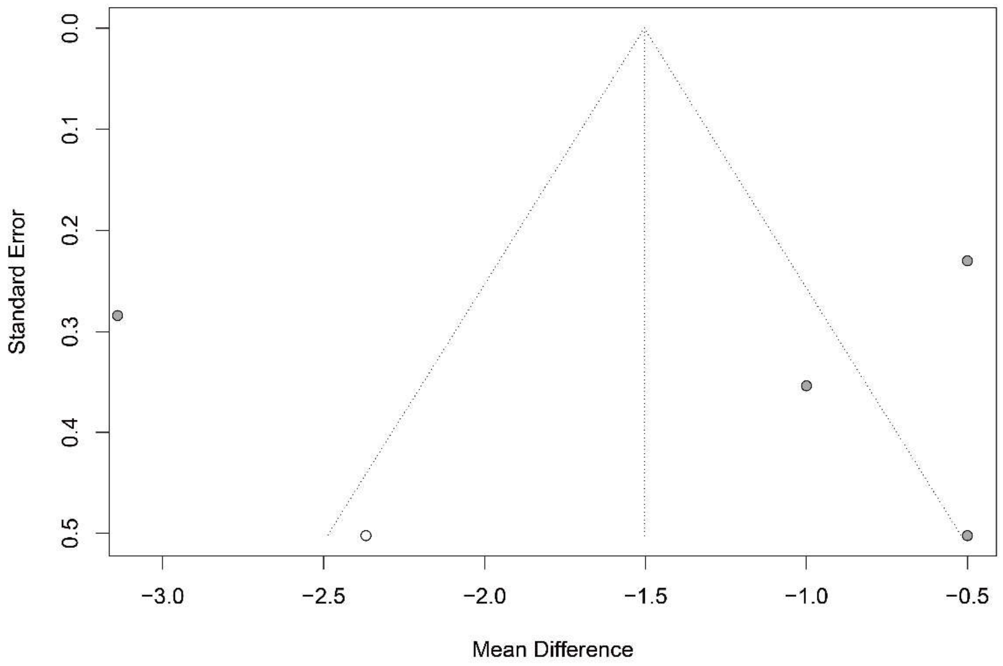

B

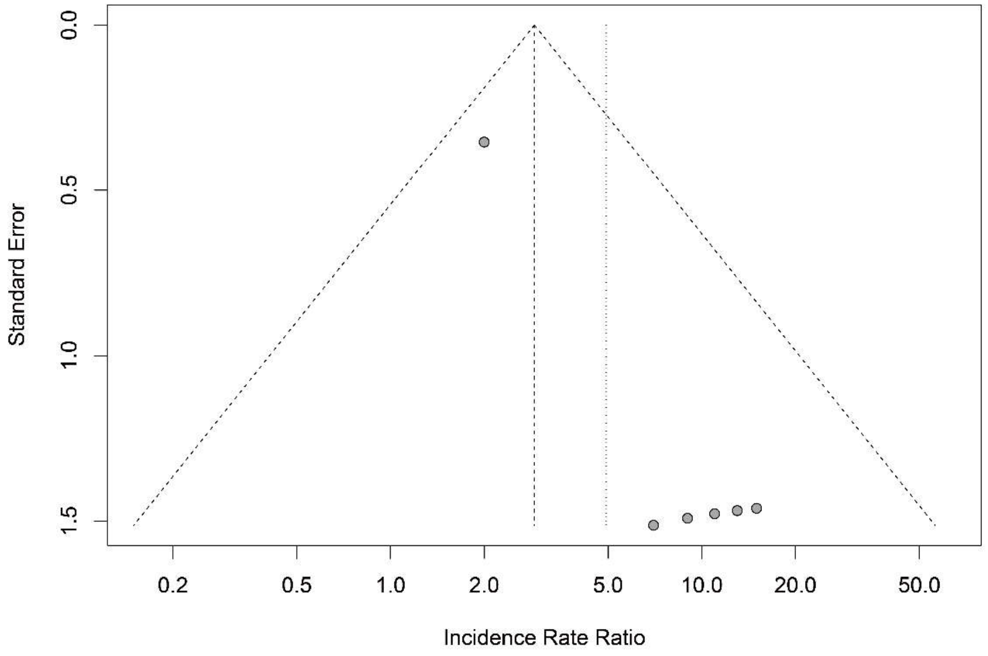

Supplementary Figure S1. Funnel plots for evaluating publication bias for intervention efficacy of processed allergen/meat from fish and crustacea on OIT. (A) Trim and filled methods for included case-control studies in mouse models. Note: Leung *et al.* (2017) study was excluded from publication bias analysis as the standard deviation of anaphylactic score after OIT treatment is zero. (B) Included cohort studies in clinical patients.
